# Successful Treatment of Post-tuberculosis Pulmonary Aspergillosis With Liposomal Amphotericin B in a Patient After a Rare Event of Voriconazole-Associated Hypotension: A Case Report

**DOI:** 10.7759/cureus.87491

**Published:** 2025-07-07

**Authors:** Ali Alsaeed

**Affiliations:** 1 Infectious Disease Division, Internal Medicine Department, Dammam Medical Complex, Dammam, SAU

**Keywords:** case report, hypotension, liposomal amphotericin b, pulmonary aspergillosis, voriconazole

## Abstract

Pulmonary aspergillosis (PA) is a serious lung infection caused by *Aspergillus* species, primarily affecting individuals with structural lung abnormalities. Common risk factors include pulmonary tuberculosis (TB) and other chronic lung diseases. Voriconazole, a second-generation triazole, is the preferred first-line treatment for invasive PA, although few adverse events are reported. Liposomal amphotericin B (LAmB) serves as an alternative treatment, particularly in cases of azole resistance or intolerance. This case report describes a rare occurrence of probable voriconazole-associated hypotension in a 52-year-old immunocompetent male with post-TB aspergillosis. The patient, previously treated for TB, presented with pleuritic chest pain, productive cough, and hemoptysis. Initial treatment with voriconazole led to significant symptomatic relief but was complicated by persistent hypotension, despite normal blood parameters. A multidisciplinary team identified voriconazole as the cause of hypotension, and treatment was switched to LAmB. Consequently, his blood pressure stabilized, and the PA symptoms resolved without any adverse events. This case underscores the importance of monitoring rare side effects during voriconazole therapy and highlights LAmB as an alternative in voriconazole-intolerant scenarios and in situations where the availability of other azoles (posaconazole and itraconazole) is limited; however, further research is necessary to optimize therapeutic strategies.

## Introduction

Pulmonary aspergillosis (PA) represents a spectrum of respiratory syndromes caused by saprophytic colonization and growth of *Aspergillus* species. PA is categorized as allergic bronchopulmonary aspergillosis (ABPA), chronic PA (CPA), and invasive PA (IPA) [1,2]. These conditions mainly affect immunocompromised patients and sometimes immunocompetent individuals with structural lung anomalies [1]. Among these, IPA is a serious fungal infection that mainly affects individuals with suppressed immunity, such as organ transplant recipients, patients with hematologic malignancies, or those undergoing prolonged corticosteroid therapy [3]. Current estimates from 2019 to 2021 indicate that over 2.1 million people develop IPA annually [4]. The crude annual mortality rate is 85.2%, primarily affecting high-risk groups [4]. Primary risk factors include pulmonary tuberculosis (TB), chronic lung diseases, emphysema, and surgically treated lung cancer [5]. TB causes irreversible morphological changes in the lung, fostering *Aspergillus* growth in the residual lung cavities [1]. This can lead to persistent symptoms, even after successful anti-TB treatment, that mimic smear-negative pulmonary TB [6], often resulting in misdiagnosis as TB reinfection or reactivation [7].

Three major classes of antifungal drugs approved for treating PA include triazoles, including voriconazole, posaconazole, isavuconazole, and itraconazole; echinocandins such as anidulafungin, caspofungin, and micafungin; and polyenes such as amphotericin B formulations [8]. Voriconazole, a second-generation triazole with good bioavailability, is the primary treatment for IPA due to its well-established safety and tolerability profile [1,8]. Common side effects include headaches, dizziness, diarrhea, and fatigue with occasional ocular disturbances, thrombocytopenia, hepatic abnormalities, and skin rashes [9]. Rarely, periostitis, encephalopathy, and hallucinations occur [10]. Severe hypotension linked to voriconazole therapy is a rare adverse event, with limited reports in the literature. A similar case was reported in an immunosuppressed individual with chronic obstructive pulmonary disease (COPD) [11]. Thus, as an alternative to voriconazole and other azoles, liposomal amphotericin B (LAmB), a modified formulation of the broad-spectrum antifungal agent amphotericin B (encapsulated in liposomes having minimal renal toxicity) is often used to treat serious and invasive fungal infections, particularly caused by fungal resistance to other antifungal agents [12]. In areas with a high prevalence of azole-resistant *Aspergillus*, LAmB is the first drug to combat adversities [8].

This case report presents a rare adverse event of probable voriconazole-associated hypotension in an immunocompetent patient with post-TB aspergillosis, subsequently treated successfully with LAmB.

## Case presentation

A 52-year-old male inmate with a history of pulmonary TB (treated two years back) was transferred to our hospital from a peripheral hospital for further evaluation. After completing a nine-month course of anti-TB treatment and testing negative for human immunodeficiency virus (HIV), he had been in good health for the past two years, until a few weeks before his hospitalization, he developed pleuritic chest pain, a productive cough with yellowish sputum, shortness of breath, and on-off hemoptysis. He denied symptoms of anorexia, weight loss, night sweats, or fever.

**Figure 1:**
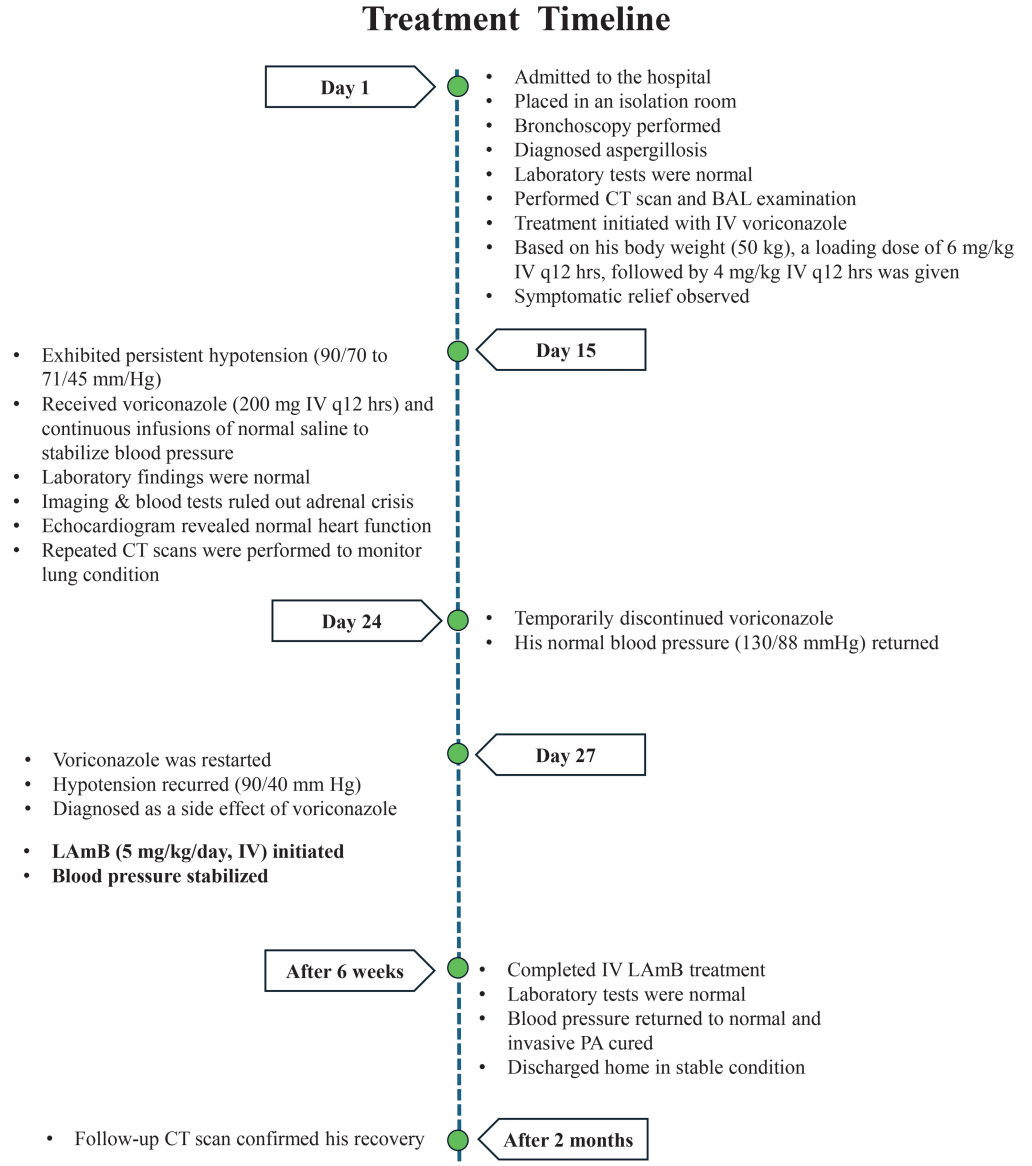
Timeline of key events and their subsequent management in this case report BAL: Bronchoalveolar lavage; CT: Computed tomography; IV: Intravenous; LAmB: Liposomal amphotericin B; PA: Pulmonary aspergillosis

**Figure 2:**
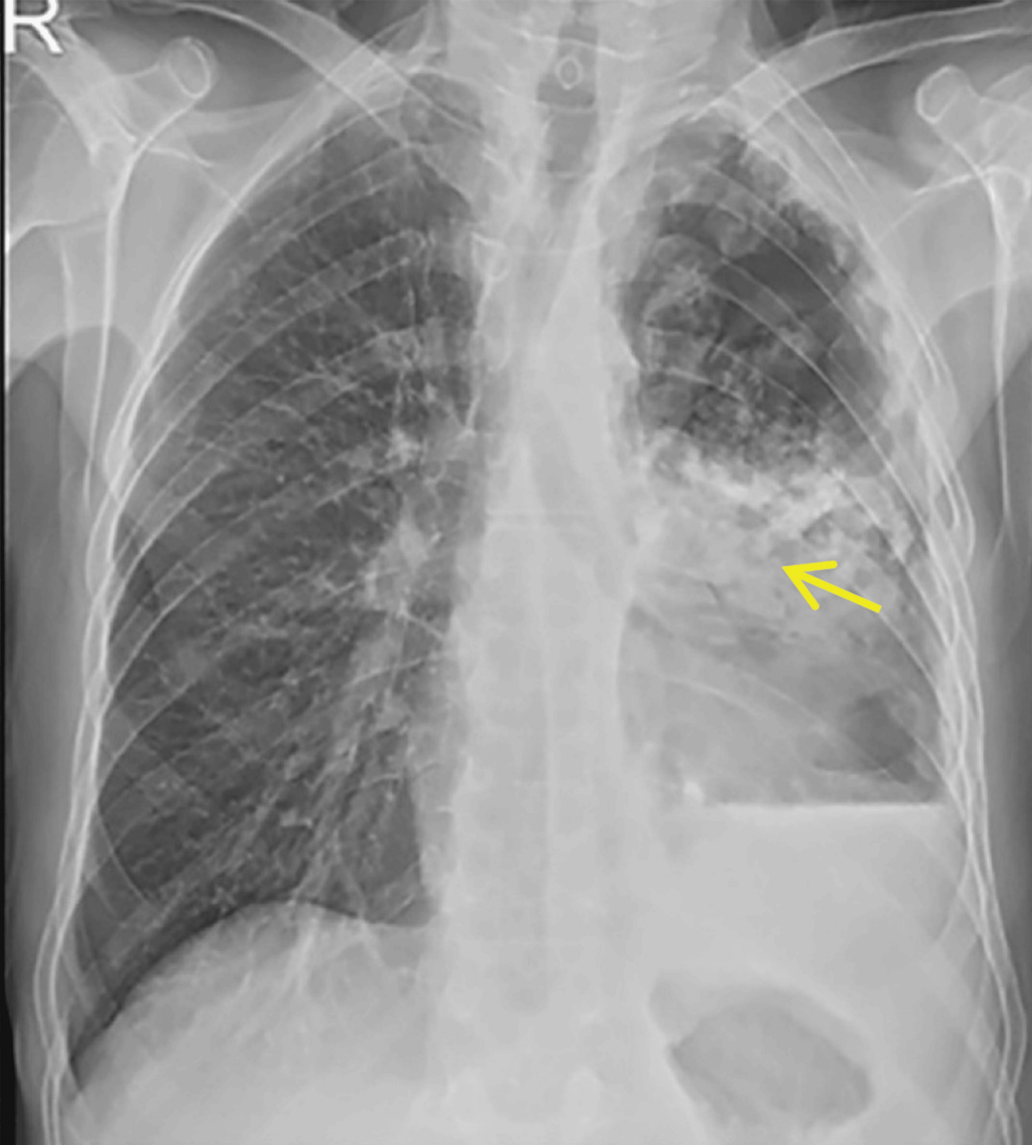
Chest X-ray on hospital admission Chest X-ray showed large air-containing cavitary lesions (indicated by yellow arrow) replacing the collapsed left lung, with intracavitary growths, suggestive of secondary fungal infection.

**Figure 3:**
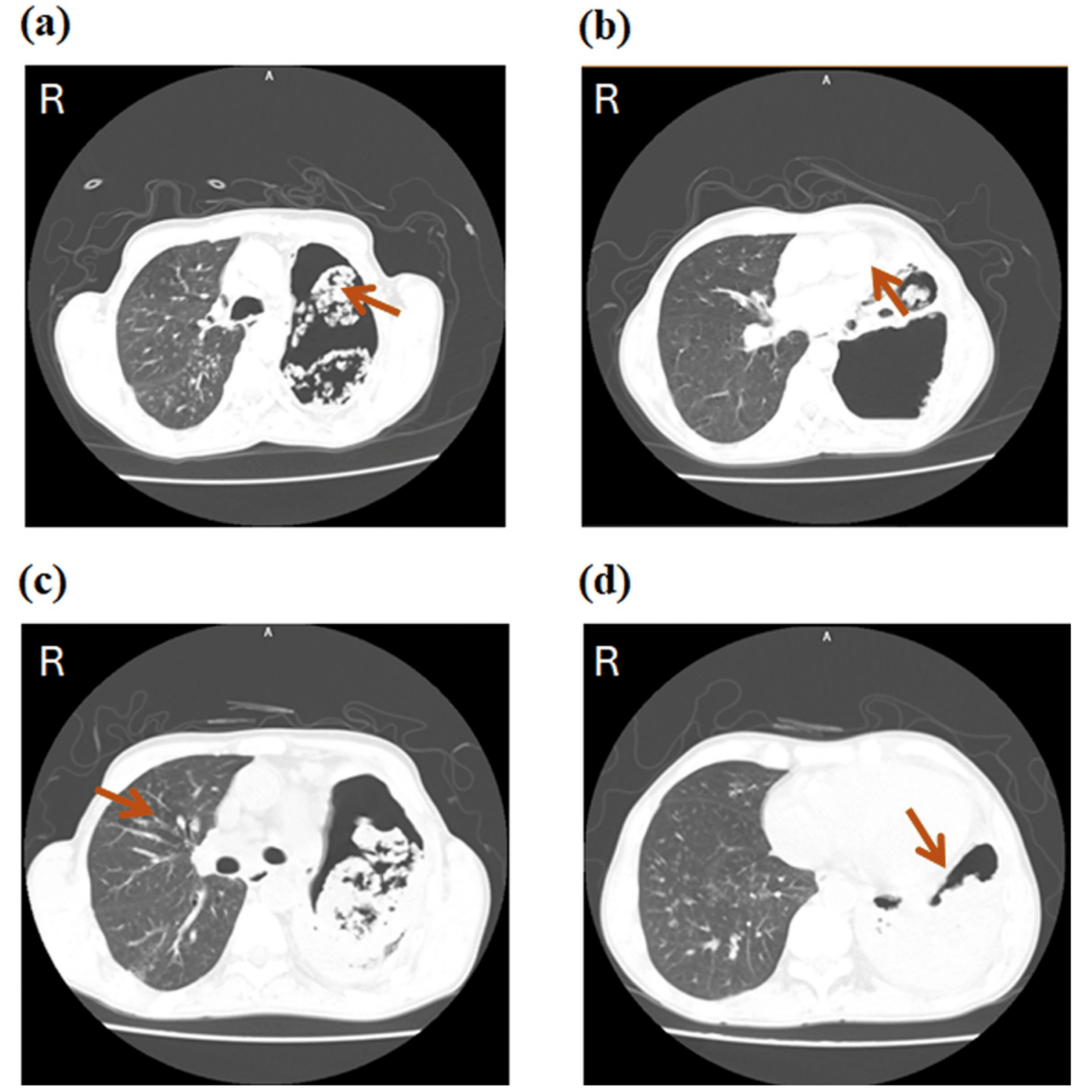
Chest CT scan in the axial plane (a) Large air-containing cavitary lesions (indicated by red arrow), occupied the whole left hemithorax, compressing the residual left lung parenchyma with a possible third lesion in the inferior lingula. (b) These cavitary lesions intercommunicated, with suspected communication with the left upper and lower lobe bronchioles; the upper lobe cavity demonstrated internal bizarre-shaped scattered fluffy soft-tissue components (some peripheral and some floating, indicated by red arrow), emphasizing the possibility of secondary fungal infection. (c) Tree-in-bud appearance is seen in the apical, posterior, and anterior segments of the upper lobes, and the superior segment of the right lower lobe, associated with patchy peribronchovascular consolidative nodules and surrounding ground glass opacities (indicated by red arrow). (d) Minimal traction bronchiolectasis versus centrilobular emphysema was noted (indicated by red arrow).

Initially, he was admitted to a peripheral hospital, where TB reactivation was suspected, but ruled out after a sputum AFB smear test, prompting his transfer to our tertiary care unit. The timeline of key events for this case is depicted in Figure 1. Upon arrival on day one, he was placed in an isolation room, and a chest X-ray (Figure 2) and bronchoscopy led to a presumptive diagnosis of probable IPA. Unfortunately, the exact species of *Aspergillus* could not be identified due to the unavailability of specific tests within our laboratory. Hematological tests, such as complete blood counts, blood glucose levels, liver enzymes, and other parameters, were normal, and HIV, hepatitis B virus, hepatitis C virus, and cytomegalovirus (CMV) screening tests were negative (Table 1). A computed tomography (CT) scan (Figure 3) and bronchoalveolar lavage (BAL) examination further yielded positive results for *Aspergillus* species. However, due to the unavailability of galactomannan antigen and β-D-glucan assays, further confirmation of the diagnosis was not done. Based on the CT scan and BAL report, the infectious disease team recommended initiating treatment with intravenous (IV) voriconazole on the same day. Based on his body weight (50 kg), a loading dose of 6 mg/kg IV twice every 12 hours was initially administered, followed by 4 mg/kg IV every 12 hours.

**Table 1:** Laboratory parameters of the patient at baseline and post-treatment with voriconazole and LAmB ACTH: adrenocorticotropic hormone; Ag/Ab: antigen and antibody; anti-HCV Ab: hepatitis C virus antibody; ALT: alanine transaminase; AST: aspartate transaminase; BDL: below the detection limit; Ca: calcium; CK: creatinine kinase; CMV: cytomegalovirus; CRP: c-reactive protein; ESR: erythrocyte sedimentation rate; Hb: hemoglobin; HBsAg: hepatitis B surface antigen; HIV: human immunodeficient virus; IgG: immunoglobulin G; K: potassium; LDH: lactate dehydrogenase; MTB PCR: *Mycobacterium tuberculosis* polymerase chain reaction; Mg: magnesium; MRSA: methicillin-resistant *Staphylococcus aureus*; Na: sodium; NA: not available; WBC: white blood cells

Parameters	At admission	During the hypotension event	At discharge	Reference range
WBC (× 10^9^/L)	7.8	5.4	6.0	4.0-11.0
Hb (g/dL)	10.7	10.1	13.5	14.0-18.0
Platelet (× 10^9^/L)	275	365	250	150-450
Neutrophil count (× 10^9^/L)	6.2	4.1	4.5	2.0-7.5
Lymphocyte count (× 10^9^/L)	0.02	0.55	1.5	1.0-3.0
Eosinophil count (× 10^9^/L)	0.12	0.2	0.3	0.02-0.5
Monocyte Count (× 10^9^/L)	1.1	0.4	0.6	0.2-0.8
Na (mmol/L)	143	141	140	135-145
K (mmol/L)	3.5	3.4	4.0	3.5-5.0
Creatinine (µmol/L)	74	50	70	60-110
ALT (U/L)	16	12	15	10-40
Albumin (g/dL)	41	38	40	35-50
AST (U/L)	16	17.2	18	10-40
Glucose random (mmol/L)	6	7	5.5	3.9-11.1
Ca (mg/dL)	2.1	2.2	2.3	2.1-2.6
Mg (mmol/L)	0.7	0.6	0.8	0.7-1.0
LDH (IU/L)	221	189	200	120-230
CK (U/L)	72	50	60	20-200
Troponin (ng/mL)	NA	BDL	<0.01	<0.01
Cortisone (µg/dL)	NA	BDL	8 (morning)	5-25 (morning), 2-9 (evening)
Blood culture and sensitivity	NA	Negative	NA	Negative
Sputum culture and sensitivity	NA	Negative	NA	Negative
MRSA screening (nasal swab)	Negative	Negative	NA	Negative
ACTH (pg/mL)	NA	50	NA	7.2-63.3
CRP (mg/L)	29	20	<5	<5
ESR (mm/h)	100	70	<20	<20 (females), <15 (males)
HIV Ag/Ab screening	Negative	NA	NA	Negative
Hepatitis B virus (HBsAg) screening	Negative	NA	NA	Negative
Hepatitis C virus (anti-HCV Ab) screening	Negative	NA	NA	Negative
CMV IgG	Negative	NA	NA	Negative
Acid fast bacteria (smear and culture)	Negative	Negative	Negative	Negative
MTB PCR	Negative	Negative	Negative	Negative

The patient responded well with significant symptomatic relief. The optimal therapeutic drug level of voriconazole was 3 mg/L, obtained approximately four hours post-dose on day four of therapy. However, by the second week (day 15) of voriconazole treatment, he exhibited persistent hypotension (90/70 mmHg to 71/45 mmHg). Despite receiving voriconazole (200 mg IV every 12 hours, based on his body weight) and continuous infusions of normal saline, his blood pressure dropped when fluids were stopped (90/40 mmHg). Electrolytes, creatinine, glucose, and liver function tests were normal (Table 1).

The patient did not experience fever, hemoptysis, dizziness, melena, diarrhea, or other notable complaints during treatment. A multidisciplinary team (comprising infectious disease specialists, endocrinologists, cardiologists, and internists) ruled out adrenal crisis after imaging and blood tests (adrenocorticotropic hormone: 2 pmol/L, cortisol (morning): 200 nmol/L, renal function tests: normal). An echocardiogram revealed normal heart function, and repeated chest CT scans were performed to monitor lung condition. However, the cause of his persistent hypotension remained inconclusive. Due to deteriorating blood pressure, voriconazole was temporarily discontinued towards the end of the third week (day 24), resulting in normalized blood pressure of 130/88 mmHg. However, the hypotension recurred when voriconazole was reintroduced after three days (day 27). This led to the diagnosis that the patient was experiencing hypotension as a side effect of voriconazole, despite maintaining a therapeutic drug level (3-4 mg/L).

**Figure 4:**
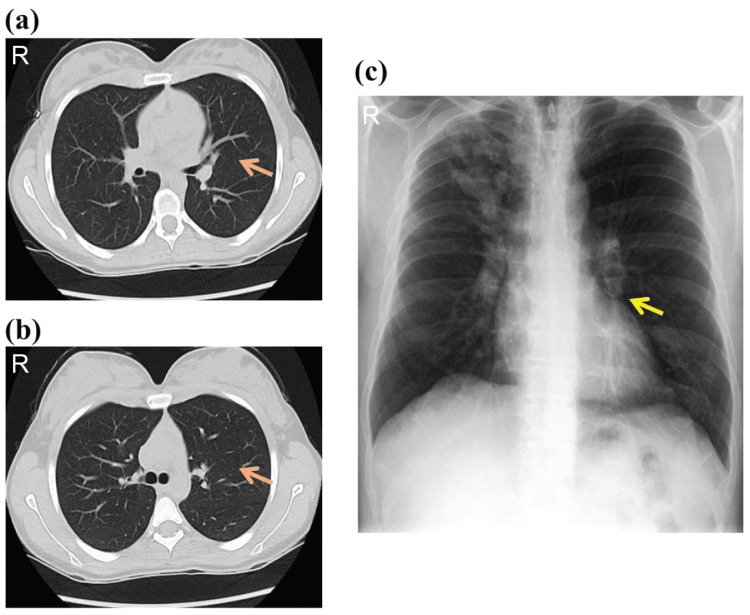
Imaging analysis during follow-up (a, b) Chest CT scan in the axial plane showed no residual air-containing cavitary lesions (indicated by pink arrow) in the left hemithorax, no compression of the left lung parenchyma, no evidence of previously suspected third cavitary lesion in the inferior lingula; previously noted tree-in-bud appearance resolved in the apical, posterior, and anterior segments of the upper lobes, as well as the superior segment of the right lower lobe, no patchy peribronchovascular consolidative nodules or surrounding ground-glass opacities; no evident traction bronchiolectasis or centrilobular emphysema, normal high-resolution CT with no residual findings related to previous aspergillosis (complete resolution of prior abnormalities). (c) Chest X-ray on follow-up showed complete resolution of the previously noted large air-containing cavitary lesions (indicated by yellow arrow), no evident intracavitary growth, fully expanded left lung with no signs of collapse, no abnormalities or residual signs of fungal infection in the left lung or any other part of the chest, no residual signs of secondary fungal infection (indicating successful treatment and recovery).

Consequently, treatment was switched to IV LAmB (5 mg/kg/day) as an alternative therapy. The manufacturing company for voriconazole was informed about the adverse effects. With LAmB, the patient’s blood pressure stabilized and remained within his baseline range, with no further recurrence of hypotension. Laboratory tests were repeated and exhibited normal ranges (Table 1). The patient was discharged in stable condition after six weeks of IV LAmB treatment. A follow-up CT scan and chest X-ray two months later, at the Infectious Disease Division outpatient clinic, confirmed his recovery (Figure 4).

## Discussion

Secondary fungal infections in the lungs pose serious global health concerns in individuals with chronic respiratory diseases, such as COPD and TB, as the damaged lung tissue provides an ideal environment for fungal growth [1]. The global prevalence of PA post-COPD is 7.39%, while in the United Arab Emirates (UAE), it is reportedly low at 1.9% [13]. In the UAE, the incidence of IPA post-COPD is estimated at 5.9 cases per 100,000 individuals [14]. Following TB, PA is more frequent and affects approximately 1.2 million people globally [1], with many cases misdiagnosed as TB reinfection or reactivation [7]. The prevalence of PA post-TB in the Middle East remains unclear [15].

Voriconazole is the standard treatment for IPA, approved by the United States Food and Drug Administration, as well as the European Medicines Agency since 2002 for adults and pediatric patients (≥12 years) [9]. Long-term oral voriconazole administration (3 to 4 mg/kg twice a day) is recommended for the treatment of post-TB aspergillosis [8]. It functions as a substrate and isoenzyme (CYP2C9, CYP2C19, and CYP3A4) antagonist for membrane transporters [9,16]. It metabolizes hepatically, with nonlinear pharmacokinetics, and has a half-life of six hours [16]. Plasma concentrations and systemic exposure to voriconazole vary among individuals influenced by hepatic phenotype, immune status, inflammation, and age, affecting its metabolism [16].

In the present case, a presumptive diagnosis of IPA was made, and the patient was treated with voriconazole. He initially responded well to voriconazole; however, after a few days, he experienced unexplained hypotension. This is a rare occurrence, and an extensive literature search revealed a similar case of hypotension due to voriconazole in an immunosuppressed patient with COPD on frequent courses of oral steroids [11]. One proposed mechanism suggests that imbalanced dose-dependent elimination of voriconazole may lead to its increased plasma levels, which in turn could result in the accumulation of histamine blockers and increase histamine release, as observed with certain azole antifungals [17]. Eventually, this could have led to vasodilation and subsequent hypotension [18]. Drug-drug interaction could also be a possibility since voriconazole's functioning has been reported to have a potentially hazardous interaction with certain concomitant medications [1]. Our case reports probable voriconazole-associated hypotension; however, its mechanism remains unclear, highlighting the need for regular blood pressure monitoring by healthcare professionals while administering voriconazole to patients.

In our case, the switch in therapy from voriconazole to LAmB helped resolve the hypotension and provided symptomatic relief to the patient. Recently, LAmB has shown promise in treating severe fungal infections as an alternative therapy of choice, showing a 61% clinical response to post-TB aspergillosis without hepatic and renal impairment [1]. The rise of azole resistance, potential drug-drug interactions, and related toxicities often restrict the use of azoles for treating PA. In cases where azoles are contraindicated, or not tolerated, or not available at the time of treatment (as in the present case), LAmB serves as a viable option for an alternative therapy [8]. It has consistently proven its efficacy in treating PA with severe complications. Intratracheal instillation of LAmB was successful in treating a Japanese patient with invasive tracheobronchial-PA [19]. Moreover, in immunocompromised patients, administering 3 mg/kg/day LAmB is recommended as a first-line treatment of PA [20]. Systemic LAmB treatment for IPA reported a few adverse events, including infusion-associated toxicity (primarily fever, chills, and hypoxemia), renal toxicity, anemia, and hypokalemia [8]. However, in our case, LAmB treatment for post-TB PA exhibited a favorable outcome for the patient without any severe adverse events.

Although adverse events such as hypotension are rare, clinicians need to be more cautious while using voriconazole. Typically, other azoles, such as posaconazole and itraconazole, are generally preferred alternatives to voriconazole due to their favorable safety profile and oral availability, but these were unavailable at our center and nearby referral facilities during the treatment period, thereby limiting our options for antifungal therapy. Although caspofungin could be considered in this situation, based on the clinical severity and the treating team’s experience, LAmB was selected due to its broad-spectrum coverage and efficacy in IPA despite its cost and toxicity profile. Hence, LAmB was administered out of clinical necessity, rather than as a preferred first-line alternative. Nonetheless, successful management of the patient with LAmB shows its promise as an alternative therapy in voriconazole-intolerant patients suffering from PA post-TB, possibly stemming from drug-drug interactions and adverse side effects, including visual disturbances, liver enzyme anomalies, neurotoxicity, and hypersensitivity reactions. Furthermore, the involvement of a multidisciplinary team facilitated a thorough diagnostic evaluation and decision-making.

While this case report provides valuable insights into adverse drug reactions and an alternative treatment strategy, few limitations exist. The specific *Aspergillus* species could not be identified due to laboratory constraints (lack of specialized equipment and reagents required for detailed fungal species identification). The absence of fungal species identification represents a significant limitation in the diagnostic workup, as it may influence therapeutic decision-making. Different *Aspergillus* species can exhibit variable susceptibility to antifungal agents, particularly with rising reports of triazole resistance in non-fumigatus species such as A. *terreus* or A. *flavus*. Furthermore, due to the unavailability of galactomannan antigen and β-D-glucan assay at the center, these tests could not be performed to further confirm the diagnosis. These assays are crucial in the appropriate diagnosis and timely management of fungal infections. Further research and broader case studies are necessary to validate these findings and optimize therapeutic decisions in similar clinical settings.

## Conclusions

This case highlights a rare adverse effect of probable voriconazole-associated hypotension in a patient with post-TB PA. The hypotension resolved on drug withdrawal and recurred upon reinitiation. Although voriconazole is the standard treatment for this infection, this case shows the importance of closely monitoring patients for side effects. When the patient developed persistent low blood pressure, switching to LAmB proved to be an effective and well-tolerated alternative. This demonstrates that LAmB may be considered a good alternative in select cases of voriconazole intolerance or when first-line agents are unavailable.
